# Citrin-deficient patient-derived induced pluripotent stem cells as a pathological liver model for congenital urea cycle disorders

**DOI:** 10.1016/j.ymgmr.2024.101096

**Published:** 2024-05-30

**Authors:** Mai Okano, Masahiro Yasuda, Yui Shimomura, Yoshikazu Matsuoka, Yasumasa Shirouzu, Tatsuya Fujioka, Masatoshi Kyo, Shoji Tsuji, Kazunari Kaneko, Hirofumi Hitomi

**Affiliations:** a Department of iPS Stem Cell Regenerative Medicine, Kansai Medical University, Osaka, Japan; b Department of Pediatrics, Kansai Medical University, Osaka, Japan; c Department of Neuropsychiatry, Kansai Medical University, Osaka, Japan

**Keywords:** Induced pluripotent stem cells, Citrin deficiency, Urea cycle disorder, Ammonia, Sodium pyruvate

## Abstract

Citrin deficiency is a congenital secondary urea cycle disorder lacking useful disease models for effective treatment development. In this study, human induced pluripotent stem cells (iPSCs) were generated from two patients with citrin deficiency and differentiated into hepatocyte-like cells (HLCs). Citrin-deficient HLCs produced albumin and liver-specific markers but completely lacked citrin protein and expressed argininosuccinate synthase only weakly. In addition, ammonia concentrations in a medium cultured with citrin-deficient HLCs were higher than with control HLCs. Sodium pyruvate administration significantly reduced ammonia concentrations in the medium of citrin-deficient HLCs and slightly reduced ammonia in HLCs differentiated from control iPSCs, though this change was not significant. Our results suggest that sodium pyruvate may be an efficient treatment for patients with citrin deficiency. Citrin-deficient iPSCs are a pathological liver model for congenital urea cycle disorders to clarify pathogenesis and develop novel therapies.

## Highlights

• Human induced pluripotent stem cells (iPSCs) were generated from two citrin deficiency (CD) patients.
• CD-iPSCs were differentiated into hepatocyte-like cells (CD-HLCs).
• CD-HLCs showed higher levels of ammonia in the culture medium compared to controls.
• Sodium pyruvate reduced ammonia concentrations in the medium of CD-HLCs.
• CD-iPSCs are a pathological model for congenital urea cycle disorders to clarify pathogenesis and develop novel therapies.

## 1 Introduction

Citrin deficiency is caused by sequence variants of the *SLC25A13* gene on chromosome 7q21.3, which encodes citrin, a calcium-binding mitochondrial solute carrier protein [1]. Citrin is a liver-type calcium (Ca^2+^)-stimulated aspartate-glutamate carrier (AGC) that mediates the electrogenic exchange of mitochondrial aspartate for cytosolic glutamate and protons [2]. AGCs provide aspartate for the synthesis of urea, proteins, and nucleotides. In addition, AGCs contribute to gluconeogenesis from lactate and transport cytosolic nicotinamide adenine dinucleotide (NADH) reducing equivalents into mitochondria as part of the malate-aspartate shuttle [3,4]. Citrin deficiency comprises both adult-onset type II citrullinemia (CTLN2) and neonatal intrahepatic cholestasis by citrin deficiency (NICCD) [3], which causes jaundice with intrahepatic cholestasis, growth retardation, hypoproteinemia, hypoglycemia, and multiple amino acidemia including citrullinemia [[5], [6], [7]]. Most patients undergo spontaneous remission within 6–12 months of an appropriate treatment of medium-chain triglyceride milk and fat-soluble vitamin supplementation [8]. <10% of patients with citrin deficiency are estimated to progress to CTLN2 [3], which is characterized by recurrent attacks of hyperammonemia, citrullinemia, liver steatosis, and neuropsychiatric symptoms, including disorientation delirium, mental derangement, sudden unconsciousness [9,10]. Some patients develop severe liver dysfunction that requires liver transplantation, which is remarkably effective; other curative treatments have not yet been fully developed [[11], [12], [13]].

The lack of useful research tools (e.g., in vitro and in vivo models) makes therapeutic development difficult. On the other hand, human induced pluripotent stem cells (iPSCs) are promising because these cells proliferate indefinitely and differentiate into specific cell lineages [14]. Hepatocytes differentiated from iPSCs have been used as models for several liver-related diseases [15], but there have been few studied in patients with citrin deficiency.

In this study, we aimed to establish in vitro models of urea cycle disorders using patient-specific human iPSCs to mimic specific pathological conditions. Human iPSCs were generated from two patients with citrin deficiency and differentiated into hepatocyte-like cells (HLCs). In addition, we compared iPSCs derived from patients with citrin deficiency to those from control. HLCs differentiated from citrin-deficient patient-specific iPSCs exhibited greater concentrations of ammonia than control iPSCs. Patient-derived citrin-deficient iPSCs can be used as a pathological liver model for congenital urea cycle disorders.

## 2 Methods

### 2.1 Human iPSC culture

Blood samples were taken from two patients with citrin deficiency (CD1 and CD2), and iPSCs were generated as described below. This study was authorized by the ethics committee of Kansai Medical University (approval number: 2021067), and written consent was obtained from the donors. All experiments were carried out in compliance with relevant institutional guidelines and the principles of the 2013 revised Declaration of Helsinki. Peripheral blood mononuclear cells (PBMCs) were isolated using Ficoll solution (Merck KGaA, Darmstadt, Germany) and were reprogrammed into iPSCs. Episomal vectors encoding human factors (OCT3/4, SOX2, KLF4, L-MYC, LIN28, mp53DD and EBNA; addgene, MA, USA) were transfected into PBMCs by electroporation. The cells were cultured on SNL feeder cells treated with mitomycin C (Fujifilm, Osaka, Japan). After 16–25 days, iPSC colonies were individually selected. These cells were cultured in DMEM/Ham's F-12 medium (Fujifilm) supplemented with 10 μg/mL fibroblast growth factor 2 (FGF2, Fujifilm), 20% knockout serum replacement (KSR, Thermo Fisher Scientific, MA, USA), 1% non-essential amino acids (Thermo Fisher Scientific), 55 mM 2-mercaptoethanol (Thermo Fisher Scientific), and 50 U/mL penicillin/streptomycin (Thermo Fisher Scientific).

To obtain feeder-free iPSCs, SNL feeder cells were enzymatically removed using CTK solution consisting of 0.1% collagenase IV, 0.25% trypsin, 20% KSR, and 1 mM CaCl_2_ in PBS (Worthington Biochemical, NJ, USA). The iPSC colonies were detached using Accutase (Innovative Cell Technologies, CA, USA), pipetted to obtain single cells, and seeded into a dish as follows. The cells (1 × 10^5^ cells) were seeded into a 6-cm dish coated with iMatrix-511 silk (Matrixome, Osaka, Japan) and incubated with NutriStem hPSC XF medium (Sartorius AG, Göttingen, Germany) complemented with 5 μM Y-27632 (Focus Biomolecules, PA, USA) for 1 day at 37 °C in a 5% CO_2_ incubator. Thereafter, the medium was changed daily with NutriStem hPSC XF without Y-27632. Cells were passaged when the colonies reached 70%–90% confluency.

Normal human iPSCs (585A1, cell number HPS0354, male, 30's, Japanese) were provided by the RIKEN BRC cell bank and cultured in a feeder-free system as described above. HepG2 cells were maintained in DMEM/Ham's F-12 medium supplemented with 10% fetal bovine serum (GE Healthcare, IL, USA).

### 2.2 Sequence variants analysis

The sequence variants analysis was performed on two patient samples (CD1 and CD2) obtained from peripheral blood at the time of clinical diagnosis. CD1 had a compound heterozygous sequence variants of NM_014251.3:c.1177+1G>A [c.Ivs11+1G>A] and NM_014251.3:c.1801G>T [p.E601*], while CD2 had a compound heterozygous sequence variants of c.Ivs11+1G>A and NM_014251.3:c.851_854del [c.851del4]. Genomic DNA was isolated from fresh iPSCs samples using the PureLink Quick Plasmid Miniprep Kit (Thermo Fisher Scientific). The primer pairs used for the analysis of the c.Ivs11+1G>A, p.E601*, and c.854del4 sequence variants are shown in Table 1. The PCR products were sequenced by the Sanger method on an automated DNA sequencer using the BigDye Xterminator purification kit (Thermo Fisher Scientific) according to the manufacturer's instructions.

**Table 1:** Primers used in PCR analysis

	Forward	Reverse	Size (bp)
*Ivs11+1g>a*	ACTACCTTCTTCTTTCTTCTGACCT	TGATTAGCCAAGCCCTAGATGC	397
E601*	GCAGCATCTTTAGTGACCCC	GTATGTTCCCCTTGGAAATGACC	492
*851del4*	TCAGGTTACACCCATGGAAGTTG	TAAGTCAGATACCAATGCCGCAA	451
*NANOG*	CTGCTGAGATGCCTCACACG	TGCCTTTGGGACTGGTGGA	99
*OCT4*	TCTCGCCCCCTCCAGGT	GCCCCACTCCAACCTGG	220
*SOX2*	AGCTACAGCATGATGCAGGA	GGTCATGGAGTTGTACTGCA	126
*SOX1*	GAGTGGAAGGTCATGTCCGAGG	CCTTCTTGAGCAGCGTCTTGGT	136
*PDGFRα*	CCGTGGGCACGCTCTTTACTCCATGT	GGATTAGGCTCAGCCCTGTGAGAAGAC	139
*AFP*	CTTTGGGCTGCTCGCTATGA	TGGCTTGGAAAGTTCGGGTC	175
*SOX17*	GTGGACCGCACGGAATTTG	GGAGATTCACACCGGAGTCA	75
*ALB*	TTTATGCCCCGGAACTCCTTT	AGTCTCTGTTTGGCAGACGAA	148
*ASGR1*	ATGAAGTCGCTAGAGTCCCAG	CAGGTCAGACACGAACTGCTT	99
GAPDH	TGCACCACCAACTGCTTAGC	GGCATGGACTGTGGTCATGAG	87
β-actin	CTGGAACGGTGAAGGTGACA	AAGGGACTTCCTGTAACAATGCA	140

### 2.3 Karyotyping and confirmation of differentiation into germ layers

Analysis of chromosomal G-bands of CD1- and CD2-iPSCs was performed by Nihon Gene Research Institute (Miyagi, Japan). For the confirmation of differentiation into germ layers, cells were applied to a V-shaped 96-well plate and the medium was replaced every 2 days with DMEM and B27. After 12 days, cells were collected in ISOGEN II (Nippon Gene, Tokyo, Japan). RT-qPCR was then performed on ectoderm (*SOX1*), mesoderm (Platelet-derived growth factor receptor α: *PDGFRα*), and endoderm (*AFP*, *SOX17*). The primers are shown in Table 1.

### 2.4 Hepatocyte-like cell differentiation

Two patient-derived citrin-deficient iPSC lines (CD1 and CD2) and a control iPSC line (585A1) were differentiated into HLCs using previously reported stepwise protocols with some modifications [16]. To obtain single cells, iPSCs were treated with Accutase at 80%–90% confluency, dissociated, and gently pipetted. Cells were cultured with NutriStem hPSC XF medium and Y-27632 on iMatrix-511 silk-coated 24-well plates. Step 1 (4 days): Cells were cultured with RPMI 1640 medium, B27, Activin A (Fujifilm), and CHIR9902 on day 1 only. Step 2 (4 days): B27, recombinant human bone morphogenetic protein 4 (BMP4), and FGF2 were added to RPMI 1640 and cultured for 4 days. Step 3 (8–10 days): The medium was changed to hepatocyte culture medium (HCM, Lonza, Switzerland) and culturing continued for 8–10 days.

### 2.5 Reverse transcription-polymerase chain reaction (RT-PCR)

RNA was isolated from cells using the ISOGEN II reagent. Reverse transcription was performed using ReverTra Ace qPCR RT Master Mix (Toyobo, Osaka, Japan). PCR was performed using a HotStar Taq Plus Master Mix Kit (Qiagen, Venlo, Netherlands) with 1% agarose (Fujifilm). We used the followings condition for PCR: 30 cycles of 95 °C denaturation for 30 s, 57 °C annealing for 30 s, and 72 °C extension for 30 s. Quantitative RT-PCR (RT-qPCR) was performed using a Rotor-Gene Q (Qiagen) and Thunderbird SYBR qPCR Mix (Toyobo). The specific PCR primers used are listed in Table 1.

### 2.6 Western blotting

Cell lysates were prepared using RIPA buffer supplemented with protease inhibitors and then subjected to electrophoresis on an SDS polyacrylamide gel. Proteins were then transferred onto PVDF membranes for probing with primary antibodies against argininosuccinate synthetase (ASS), citrin, albumin (ALB, Bethl Laboratories, MA,USA), transthyretin (TTR, Proteintech, IL, USA), hepatocyte nuclear factor 4α (HNF4α, Santa Cruz Biotechnology, TX, USA), carbamoyl phosphate synthetase 1 (CPS1, Proteintech), ornithine transcarbamylase (OTC, Sigma-Aldrich, MO, USA) argininosuccinate lyase (ASL, Abcam, UK), arginase-1(ARG1, Cell Signaling Technology, MA, USA) and glyceraldehyde-3-phosphate dehydrogenase (GAPDH, Proteintech), followed by incubation with horseradish peroxidase-linked anti-mouse, anti-goat, or anti-rabbit IgG secondary antibodies. Specific protein bands were visualized using Pierce Western Blotting Substrate (Thermo Fisher Scientific).

### 2.7 Immunocytochemistry

Cells were fixed in paraformaldehyde for 30 min, permeabilized using Triton-X for 10 min, and blocked with Blocking One (Nacalai Tesque, Kyoto, Japan). Cells were then incubated with primary antibody overnight at 4 °C and then secondary antibody was applied for 60 min at room temperature. Nuclei were counterstained using Hoechst 33258, and stained cells were evaluated by fluorescence microscopy (BZ-9000, Keyence). The wavelengths used to image fluorescent signals are 360/40 nm for DAPI, 470/40 nm for GFP,620/60 nm for Cy5.

The catalog numbers of antibodies for immunocytochemistry used are listed in Table 2.

**Table 2:** Catalog numbers of antibodies for immunocytochemistry.

	Company	Catalog Number
Primary antibodies		
OCT4	BD Transduction Laboratories	611,202
NANOG	Cell Signaling Technology	4903
SSEA4	Cell Signaling Technology	4755
TRA-1-60	Cell Signaling Technology	4746
ALB	Bethyl Laboratories	A80-229 A
HNF4α	Santa Cruz Biotechnology	374,229
ASGR1	Santa Cruz Biotechnology	52,623
AFP	Shino Biological	12,177-R003

Secondary antibodies		
Mouse	Abcam	Ab150107
Rabbit	Abcam	Ab6717
Goat	Abcam	Ab6881

### 2.8 Ammonia measurement

HCM and ammonium chloride (NH_4_Cl) were incubated together after 15–20 days of differentiation into HLCs. Supernatants were collected after 3, 6, 24 and 48 h. Ammonia test kits (Fujifilm) were used according to the manufacturer's instructions.

### 2.9 Statistical analyses

Statistical analyses were performed using Graph Pad Prism 5. A one-way and two-way analysis of variances (ANOVA) with a Bonferroni post hoc test was used for assessment. *P*-values <0.05 were considered significant.

## 3 Results

### 3.1 Generation of citrin-deficient patient-specific iPSCs

Citrin-deficient iPSCs (CD-iPSCs) were generated from the peripheral blood of patients CD1 and CD2. CD1 was a male patient diagnosed with NICCD who presented with white stools, poor body weight gain, elevated serum ammonia levels (189 μg/dL) and bile acids, and jaundice. CD1 was a 24-year-old patient carrying the c.Ivs11+1 G>A and p.E601* compound heterozygous sequence variants. CD2 was a 48-year-old female patient treated as schizophrenia for a long time but diagnosed with CTLN2 at age 40, after being suspected by mild liver dysfunction (aspartate aminotransferase, 52 U/L; alanine aminotransferase, 52 U/L), fatty liver and hyperammonemia (234 μg/dL) and hypercitrullinemia (949.5 nmol/mL; normal range, 17.0–43.0 nmol/mL) [17]. CD2 carries the c.Ivs11+1 G>A and c.851del4 compound heterozygous sequence variants and underwent liver transplantation. The c.Ivs11+1 G>A sequence variant is located in intron 11 and is the most frequently reported sequence variant (36.3%) [18]. The second most common sequence variant (32.8%) is c.851del4, which is located in exon 9 [18]. c.IVS11+1 G>A and c.851del4 are sequence mutations found in east Asia [18], and we consider that the presence of these sequence mutations in two CD patients enhances the value of the model system we have developed.

**Fig. 1:**
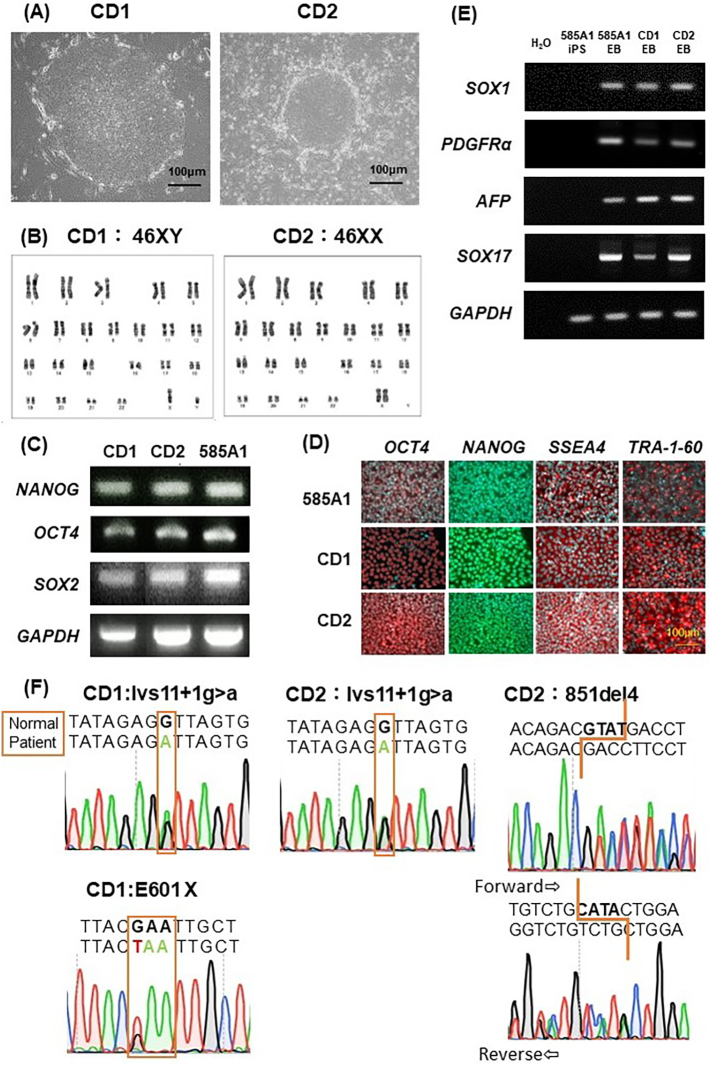
Characterization of CD-iPSCs. (A) Phase contrast images of CD-iPSCs. Magnification: 200×. Scale bars, 100 μm. (B) Normal karyotype by G-banding of CD-iPSCs. (C) Expression of pluripotency markers in CD-iPSCs. CD1-, CD2-, and 585A1-iPSCs were positive with markers *NANOG*, *OCT4*, and *SOX2*. *GAPDH* was used as a control. (D) Immunostaining for markers of pluripotency. 585A1-, CD1-, CD2-iPSCs were positive for expression of pluripotent protein such as OCT4, NANOG, SSEA4, and TRA-1-60. Scale bar, 100 μm. (E) Embryoid bodies from CD1-, CD2-, and 585A1-iPSCs formed cells of three embryonic germ layers: ectoderm (*SOX1*), mesoderm (*PDGFRα*), and endoderm (*AFP* and *SOX17*). (F) sequence variants analysis of CD-iPSCs confirmed compound heterozygous sequence variants seen in the patients.

Four iPSC-like colonies were generated from CD1 and one from CD2. One colony from each patient was selected and cloned. Both CD1-iPSCs and CD2-iPSCs exhibited normal stem cell morphology (Fig. 1A), and normal karyotypes were confirmed by G-banding analysis (Fig. 1B). The expression of pluripotency-related transcripts, including *NANOG*, *OCT4*, and *SOX2*, was confirmed by PCR (Fig. 1C). 585A1-, CD1-, CD2-iPSCs were positive for expression of pluripotent protein also such as OCT4, NANOG, SSEA4, and TRA-1-60 by immunostaining (Fig. 1D). An embryoid body-formation assay was performed, and the differentiation into three germ layers was confirmed: ectoderm (*SOX1*), mesoderm (*PDGFRa*), and endoderm (*AFP* and *SOX17*) (Fig. 1E). Taken together, these experiments demonstrate the self-renewal ability and pluripotency of CD-iPSCs. In addition, sequencing analysis confirmed that CD1-iPSC carries the c.Ivs11+1 G>A sequence variant and the p.E601* sequence variant with a GAA to TAA substitution. CD2-iPSCs carry the c.Ivs11+1 G>A sequence variant and the c.851del4 sequence variant, which causes a frameshift by the deletion of four nucleotides in exon 9 (Fig. 1F).

### 3.2 Differentiation of CD-iPSCs into hepatocyte-like cells

**Fig. 2:**
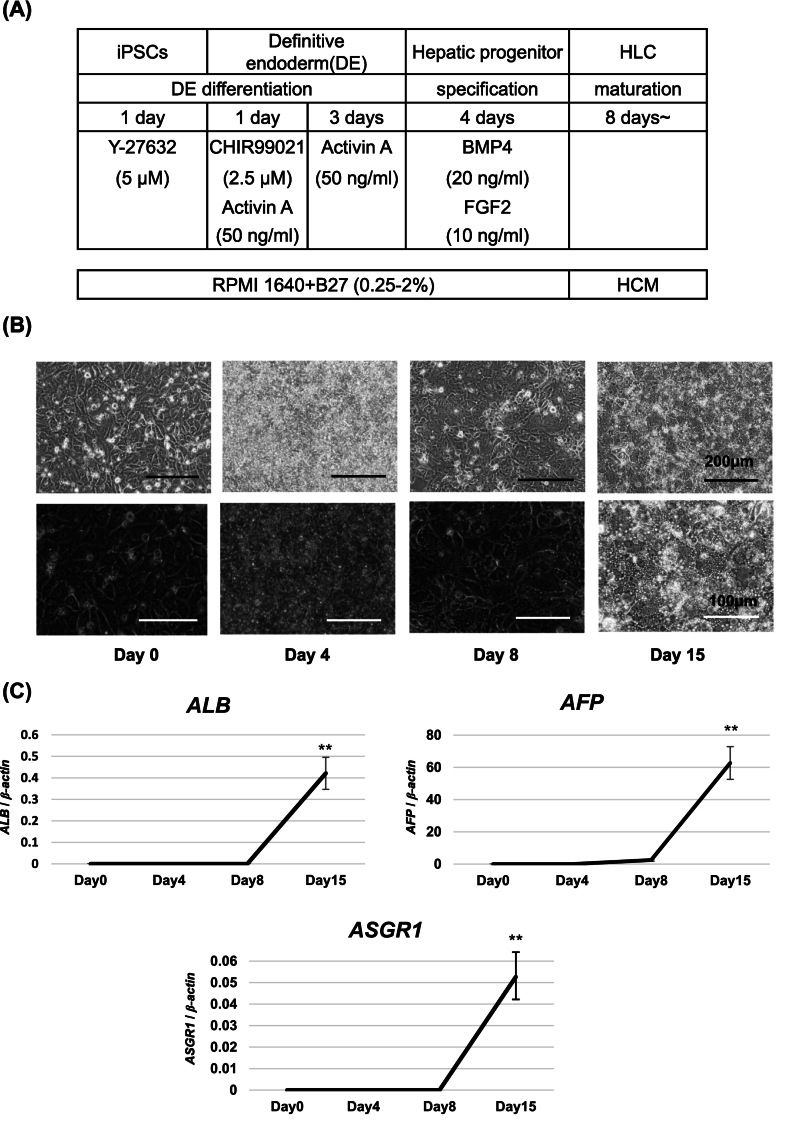
Differentiation of iPSCs into HLCs. (A) Timeline and factors involved in the differentiation of iPSCs into HLCs. The differentiation protocol was performed by modifying a previous method. (B) Phase contrast images of differentiated CD2-iPSCs. Magnification: 100× and 200×. Scale bars, 200 μm and 100 μm. (C) Expression of hepatic markers measured by RT-qPCR. *β-actin* was used as a reference gene. Results are presented as mean ± SD of 4 samples. Statistical analysis was performed using one-way ANOVA with Bonferroni's test. **, *P* < 0.01 vs undifferentiated iPSCs.

**Fig. 3:**
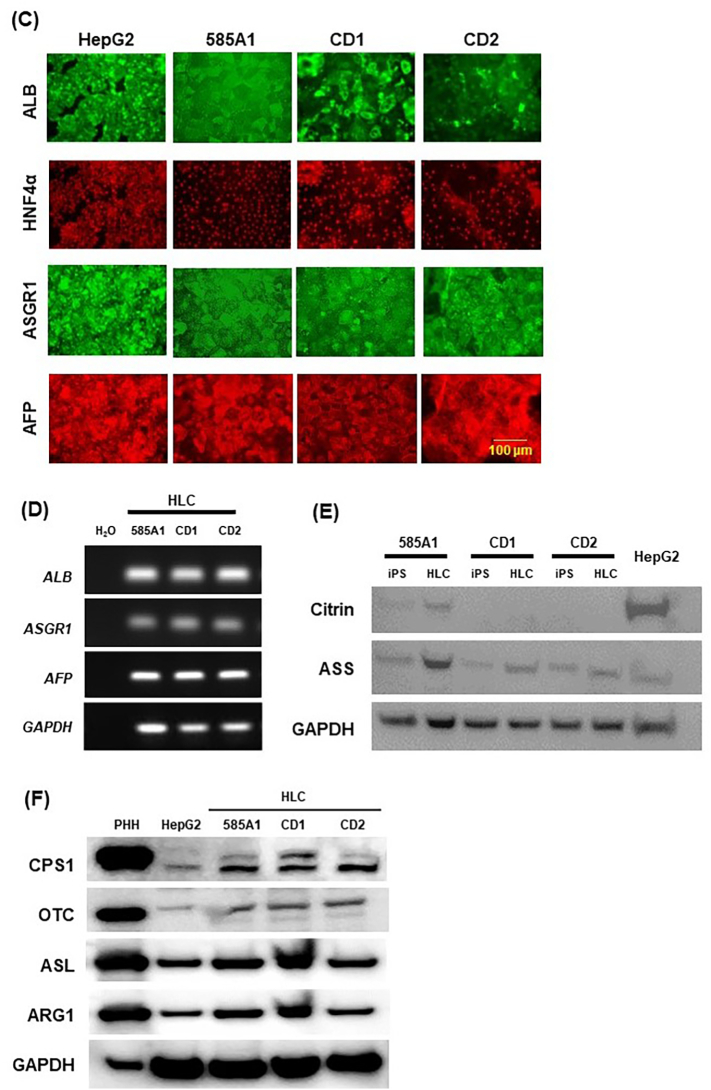
Comparison of HLCs differentiated from CD- and control iPSCs. (A) Phase contrast images of CD1-, CD2- and 585A1-HLCs. Magnification: 100× and 200×. Scale bars, 200 μm and 100 μm. (B) Immunoblot analysis for hepatic proteins in CD1-, CD2-, and 585A1-HLCs. All cells expressed ALB, TTR, and HNF4α. GAPDH was used as a control. (C) Immunostaining for markers of hepatocytes. CD1-, CD2-, and 585A1-HLCs were positive for expression of hepatic protein such as ALB, HNF4α, ASGR1, and AFP. HepG2 cells were used as a positive control for HLCs. Scale bar, 100 μm. (D) mRNA expression of hepatocyte-associated genes in HLCs. PCR showed that CD1- and CD2-, and 585A1-HLCs express ALB, ASGR1, and AFP. (E) Immunoblot analysis for citrin and ASS protein in CD1-, CD2-, and 585A1-HLCs. Citrin was not expressed in CD1- and CD2-HLCs and ASS was expressed in CD1- and CD2-HLCs more weakly than in 585A1-HLCs. GAPDH was used as a control. (F) Immunoblot analysis for CPS1, OTC, ASL, and ARG1 protein in 585A1-, CD1-, CD2-HLCs. PHH and HepG2 were used as positive controls. Enzymes of the urea cycle were expressed in 585A1-, CD1-, and CD2-HLCs. GAPDH was used as a control.

Citrin is mainly expressed in the liver and has important roles in metabolism. CD-iPSCs and 585A1-iPSCs were differentiated into hepatocytes using the protocol described previously [16] (Fig. 2A). First, we confirmed whether CD2-iPSCs could be induced into HLCs. Using our induction method, CD2-iPSCs assumed a hepatocyte-like morphology as evaluated by light microscopy (Fig. 2B), and RT-qPCR revealed expression of hepatocyte-specific markers at 15 days of CD-2HLCs (Fig. 2C). Four technical replicates were performed. Next, iPSCs were induced into HLCs. As shown in Fig. 3A, HLCs derived from two different CD-iPSCs (CD1-HLCs and CD2-HLCs) exhibited hepatocyte-like polygonal morphology similar to 585A1-HLCs. Western blotting revealed expression of liver-specific proteins such as ALB, TTR, and hepatocyte nuclear factor 4α (HNF4α) (Fig. 3B), and immunostaining also confirmed expression of ALB, HNF4α, asialoglycoprotein receptor 1(ASGR1), and AFP (Fig. 3C). The transcription of liver-specific genes (*ALB*, *ASGR1*, and *AFP*) was confirmed in both 585A1-HLCs and CD-HLCs (Fig. 3D), suggesting that wild-type HLCs and CD-HLCs exhibit a normal hepatic lineage cell phenotype. In addition, we confirmed the complete loss of citrin protein expression and the reduced expression of ASS in CD-HLCs (Fig. 3E). Western blotting revealed expression of CPS1, OTC, ASL, and ARG1 protein in 585A1-, CD1-, CD2-HLCs (Fig. 3F). PHH (primary human hepatocyte) and HepG2 were used as positive controls. Enzymes of the urea cycle were expressed in 585A1-, CD1-, and CD2-HLCs.

### 3.3 Urea cycle dysfunction in CD-HLC

**Fig. 4:**
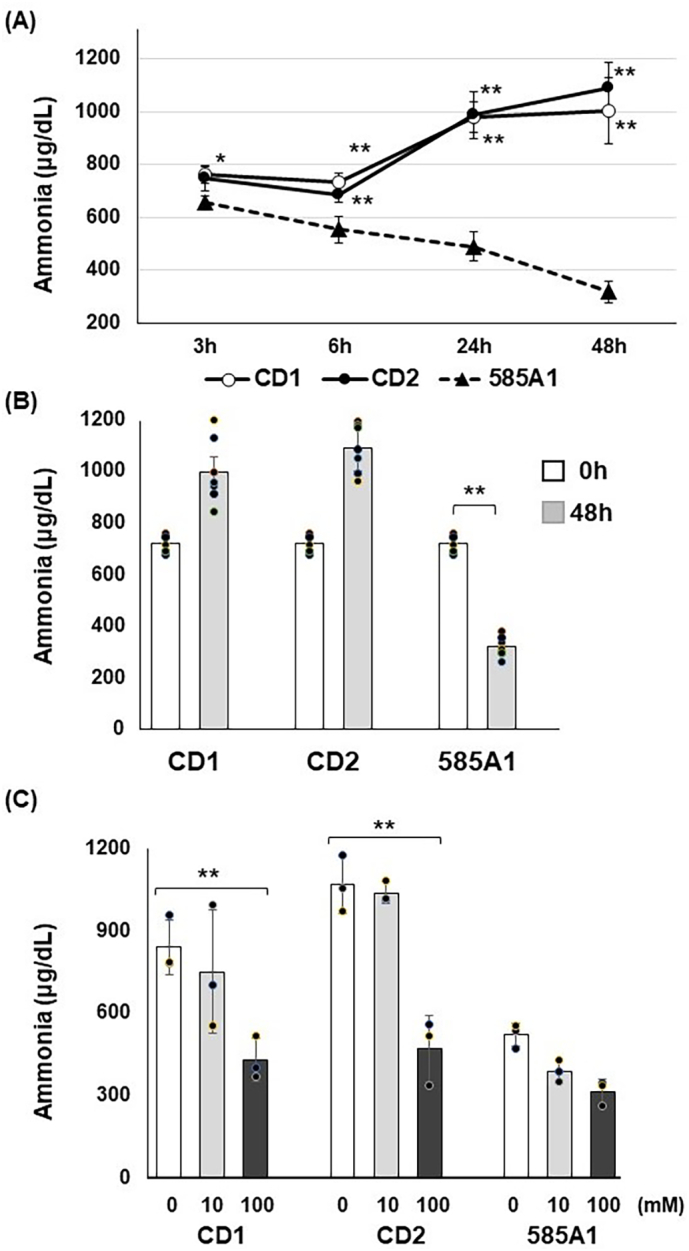
Evaluation of ammonia consumption in CD-HLCs. Each group was treated with NH_4_Cl for 48 h and ammonia concentrations were measured by absorbance spectrophotometry. (A) Ammonia concentration decreased significantly in 585A1-HLCs compared with CD-HLCs. Closed triangle, 585A1-HLCs; open circle, CD1-HLCs; closed circle, CD2-HLCs. Results are presented as means ± SD of 7 samples. Statistical analysis was performed using two-way ANOVA with Bonferroni's test. *, P < 0.01, **, *P* < 0.001585A1-HLCs vs CD-HLCs. (B) Ammonia concentration decreased significantly in 585A1-HLCs compared with CD-HLCs at 48 h. Results are presented as means ± SD of 7 samples. Statistical analysis was performed using one-way ANOVA with Bonferroni's test. **, P < 0.01 vs before NH_4_Cl treatment. (C) Effect of sodium pyruvate on ammonia consumption in HLCs. White bar without sodium pyruvate, grey bar with 10 mM sodium pyruvate, black bar with 100 mM sodium pyruvate. Results are presented as means ± SD of 3 samples. Statistical analysis was performed using one-way ANOVA with Bonferroni's test. **, P < 0.01 vs without sodium pyruvate.

Citrin deficiency is a secondary urea cycle disorder caused by a shortage of aspartate supply from mitochondria to the cytoplasm in the urea cycle [2]. To investigate whether ammonia is consumed via the urea cycle in CD-HLCs, NH_4_Cl (1 mM) was added to the medium, and the ammonia concentration in the medium, not cell lysate, was measured. After the addition of NH_4_Cl, the ammonia concentration decreased in 585A1-HLCs, indicating NH_4_Cl consumption in HLCs. However, in CD-HLCs, the ammonia concentration did not decrease, and NH_4_Cl consumption was not observed at every time point (Fig. 4A). After 48 h of NH_4_Cl treatment, ammonia concentration decreased significantly in 585A1-HLCs compared with CD-HLCs (Fig. 4B). This demonstrates a dysfunction in the urea cycle in CD-HLCs.

Sodium pyruvate is an alternative treatment for urea cycle disorders, including citrin deficiency [19]. To examine whether the addition of sodium pyruvate improves urea cycle dysfunction, HCM was supplemented with various concentrations of sodium pyruvate from day 11 to day 15 of culture, and then NH_4_Cl was added to the HCM on day 16 for 24 h of co-culture. Sodium pyruvate addition significantly reduced ammonia concentrations in a dose-dependent manner in CD-HLCs. Slight decreases were also observed in 585A1-HLCs, but these changes were not significant (Fig. 4C). Seven technical replicates were performed for Fig. 4A,B and three technical replicates were performed for Fig. 4C.

## 4 Discussion

The urea cycle is an important protein metabolism pathway in the liver that produces urea from ammonia. Urea cycle disorders are genetic diseases characterized by abnormalities in enzymes involved in this cycle, leading to conditions such as hyperammonemia. Enzymes including CPS1, OTC, ASS, ASL, and ARG1 mediate the urea cycle and comprises four amino acids: ornithine, citrulline, argininosuccinic acid, and arginine. [20]. Citrin is expressed in the liver, kidney, and heart and is involved in the transport of aspartic acid [1,21]. Citrin deficiency causes an inability to supply aspartic acid to the urea cycle, resulting in secondary urea cycle disorders [1]. In this study, iPSCs were generated from two citrin-deficient patients with different compound heterozygous sequence variants. The disease mechanism of CD is thought to be loss of citrin function. Therefore, it is highly unlikely that the variant seen in the patient is in cis. Considering this disease mechanism, plus the fact that all three sequence variants are loss-of-function, we consider that the patients in these cases are likely to be compound heterozygotes. On the other hand, CD-HLCs exhibited normal liver function regarding the production of albumin and liver-specific proteins, but also showed loss of citrin protein and decreased expression of ASS. In addition, we found that CD-HLCs exhibit decreased ammonia consumption compared to HLCs derived from control iPSCs. These abnormalities mimic the pathophysiology of citrin deficiency. Although an effective treatment for citrin deficiency has yet to be established (apart from liver transplantation), treatment with sodium pyruvate and arginine has been demonstrated clinically [19]. For instance, the administration of sodium pyruvate to CTLN2 patients reduces the frequency of encephalopathy, increases energy intake and BMI, and reduces ammonia levels [[22], [23], [24]]. Our results clarify that pyruvate administration ameliorates ammonia concentrations in CD-HLCs, suggesting that it might be an adequate treatment for patients with citrin deficiency. Based on these results, CD-HLCs have great potential as a preclinical research tool for analyzing therapeutic efficacy in vitro.

The pathophysiology of each urea cycle disorder differs depending on the site of the lesion, and the optimal treatment must be selected for each disease. For example, patients with urea cycle disorders restrict protein intake to minimize ammonia accumulation, while hyperammonemia in citrin deficiency is treated with a high-protein, low-carbohydrate diet [25,26]. Furthermore, the administration of pyruvate and arginine remains controversial for some diseases. However, there are several problems with urea cycle disorder research; patient sample collection is challenging, especially for organs such as the liver, and differences between human and mouse NADH shuttles prevent the reproduction of full pathophysiological phenotypes in citrin-deficient animal models. The absence of citrin leads to quantitative decrease of ASS and hyperammonemia, but Citrin KO mice do not show hyperammonemia and decrease of hepatic ASS activity [27]. It is possible that the high activity of glycerol-3-phosphate shuttle in mice may compensate for any decrease in malate–aspartate shuttle activity by transporting NADH-reducing equivalents back into mitochondria [31]. Double-knockout mice with disrupted mitochondrial glycerol-3-phosphate dehydrogenase (mGPD) are currently being used and manifest hyperammonemia. However, it is not clear how inhibiting the transport of reducing equivalents leads to hyperammonemia. Knocking out mGPD also may have created other metabolic dysfunctions, which limit the application of the double KO model to humans with CD [28].

In this study, HLCs were differentiated from patient iPSCs, thereby enabling the development of novel therapies and research using disease models of specific pathological conditions. Genomic repair of urea cycle sequence variants may enable cell-based clinical treatments without immunosuppression. However, this study has limitations. The iPSC-derived HLCs have been supposed to be relatively immature and resemble fetal hepatocytes rather than mature, adult hepatocytes [29]. Therefore, the possibility cannot be ruled out that an adequate urea cycle has not been formed. But Fig. 3-F shows that the expression of urea cycle enzymes is similar in both control HLCs and CD-HLCs. Another limitation is that the differentiation induction method does not reproduce liver ammonia metabolism because living hepatocytes have different metabolic functions that vary depending on their localization along the sinusoids within hepatic lobule [30]. Mature hepatocytes must be evaluated with a normal urea cycle and improved induction methods. Furthermore, this study is lacking isogenic controls which are considered the best control in such studies and all comparisons are made to a single control cell line.

Previous reports have clarified the effects of L-arginine using HLCs differentiated from citrin-deficient patient-specific iPSCs, but mild improvements in urea levels were observed [31]. On the other hand, urea production was measured using control and CD-HLCs by the method of Kim et al.; however, with less than detectable levels in our study. Therefore, we assessed the urea cycle based on ammonia concentration. The possibility of measuring urea, citrulline, and glutamine would be helpful in elucidating the disease mechanism. Moreover, sodium pyruvate significantly reduced ammonia levels in CD-HLCs. The precise mechanism by which sodium pyruvate improves the metabolic abnormalities in CD remains incompletely understood. Recent studies suggest that exogenously supplied pyruvate enter the cytoplasm, and convert to lactic acid. At this conversion, NADH is transformed into NAD^+^ and lead to a reduction in the cytosolic NADH/NAD^+^ ratio. In cases of CD, the ingestion of carbohydrates results in the metabolism of glucose through the glycolytic pathway, leading to the production of NADH and the progression of metabolic dysfunction. However, the introduction of pyruvate helps to counteract this process, alleviate glycolysis inhibition, and provide oxaloacetate and aspartate to activate cytosolic urea production [19,24,32]. Additionally, the secondary effects of pyruvate increase cellular energy metabolism [33,34], and a previous study using a liver-perfusion system in citrin knockout mice has revealed that pyruvate ameliorates ureogenesis from ammonia by reoxidizing NADH and supplying energy [35]. Although detailed mechanisms must be elucidated, our study is the first in vitro study to demonstrate the efficacy of pyruvate in patients with CD.

## 5 Conclusions

In conclusion, we investigated the pathogenesis of the urea cycle disorder using iPSCs derived from patients with citrin deficiency by inducing differentiation into HLCs. Cells derived from patients with urea cycle disorders showed inadequate ammonia metabolism compared with normal HLCs. Sodium pyruvate reduced ammonia in cells from patients with citrin deficiency, but sodium pyruvate did not alter ammonia significantly in cells from healthy subjects. These findings suggest that pyruvate may be an efficient treatment for citrin-deficient patients. These hepatic urea cycle models from human iPSCs are useful tools for studying pathogenesis and developing novel therapies for patients with urea cycle disorders.

The following is the supplementary data related to this article.

Supplemental Fig. 1 Original blots of Figures. Supplemental Fig. 1

## Authors' contributions

H·H, K·K and S.T designed the study.

M.O, M.Y, Y·S, Y.M, Y·S, T.F and H·H were involved in data acquisition and analysis.

M.O, M.Y, Y.M, Y·S, T.F, F·H, M.K, S.T, K·K and H·H analyzed and interpreted the data.

M.O and H·H wrote and revised the manuscript.

All authors read and approved the final article.

## Funding

This work was supported in part by a Grant-in-Aid for Scientific Research from the Japan Society for the Promotion of Science (21K08292), Kansai Medical University Molecular Imaging Center of Diseases, the Research Grant D2 from Kansai Medical University, the branding program as a world-leading research university on intractable immune and allergic diseases, by Katano Alumni Research Grant.

## CRediT authorship contribution statement

**Mai Okano:** Writing – original draft, Investigation, Data curation, Conceptualization. **Masahiro Yasuda:** Validation, Methodology, Data curation. **Yui Shimomura:** Investigation, Data curation. **Yoshikazu Matsuoka:** Visualization, Validation, Supervision, Methodology. **Yasumasa Shirouzu:** Validation, Supervision, Methodology, Investigation, Formal analysis. **Tatsuya Fujioka:** Validation, Supervision, Investigation, Data curation, Conceptualization. **Masatoshi Kyo:** Validation, Supervision, Investigation. **Shoji Tsuji:** Validation, Supervision, Project administration. **Kazunari Kaneko:** Supervision, Project administration, Funding acquisition. **Hirofumi Hitomi:** Writing – original draft, Supervision, Investigation, Funding acquisition, Data curation, Conceptualization.

## Declaration of competing interest

None.

## Data Availability

Data will be made available on request.
