# Supplementary material for: Citrin-deficient patient-derived induced pluripotent stem cells as a pathological liver model for congenital urea cycle disorders
Source: Mol Genet Metab Rep. 2024 May 30;40:101096. doi: 10.1016/j.ymgmr.2024.101096 (PMC11170474; doi:10.1016/j.ymgmr.2024.101096)
Supplement: Supplemental Fig. 1 — Original blots of Figures. [file mmc1.pptx]

## Slide 1
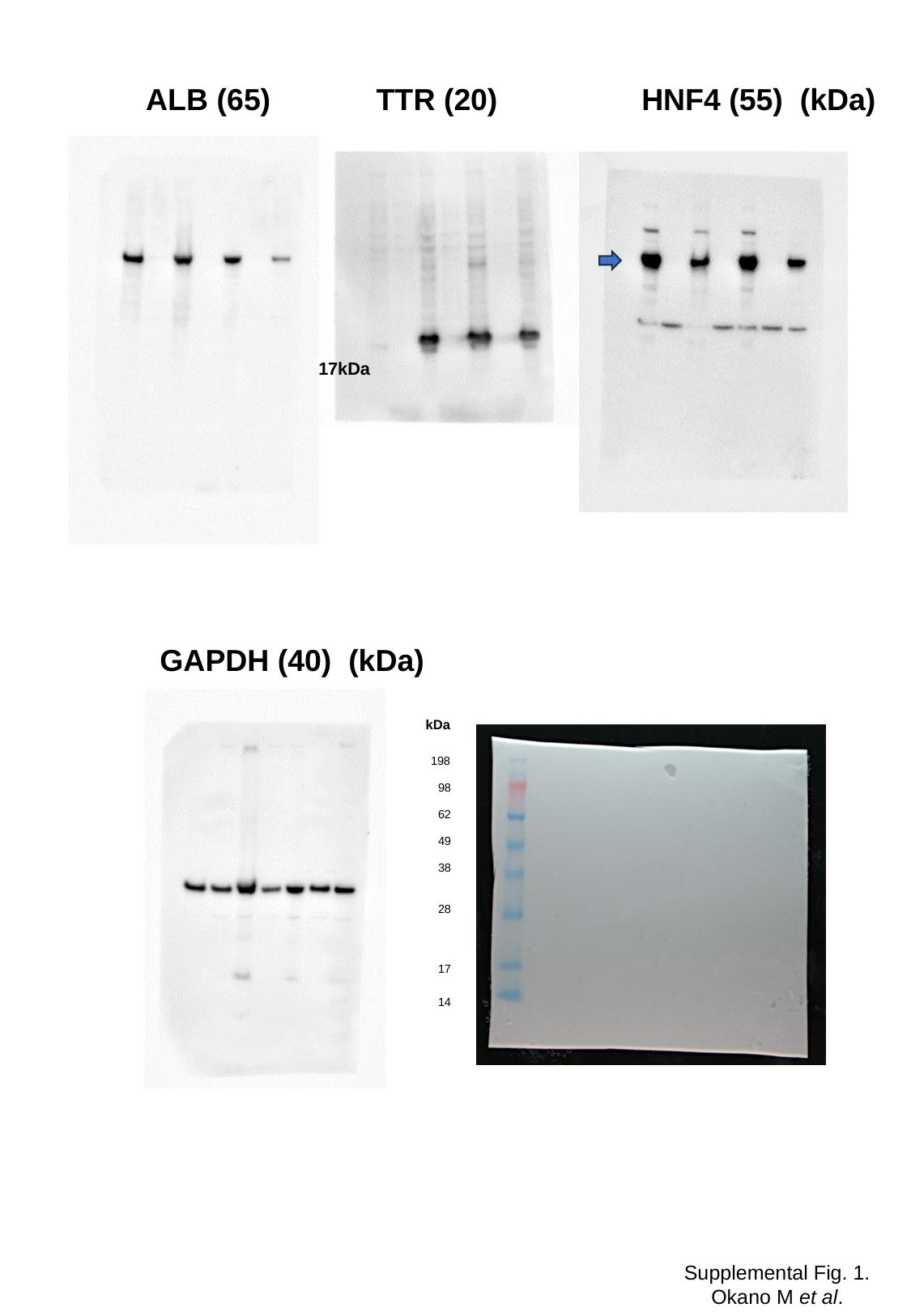

ALB (65)　　　TTR (20)　　 　　HNF4 (55) (kDa)
17kDa
GAPDH (40) (kDa)
kDa
198
98
62
49
38
28
17
14
Supplemental Fig. 1. Okano M et al.

## Slide 2
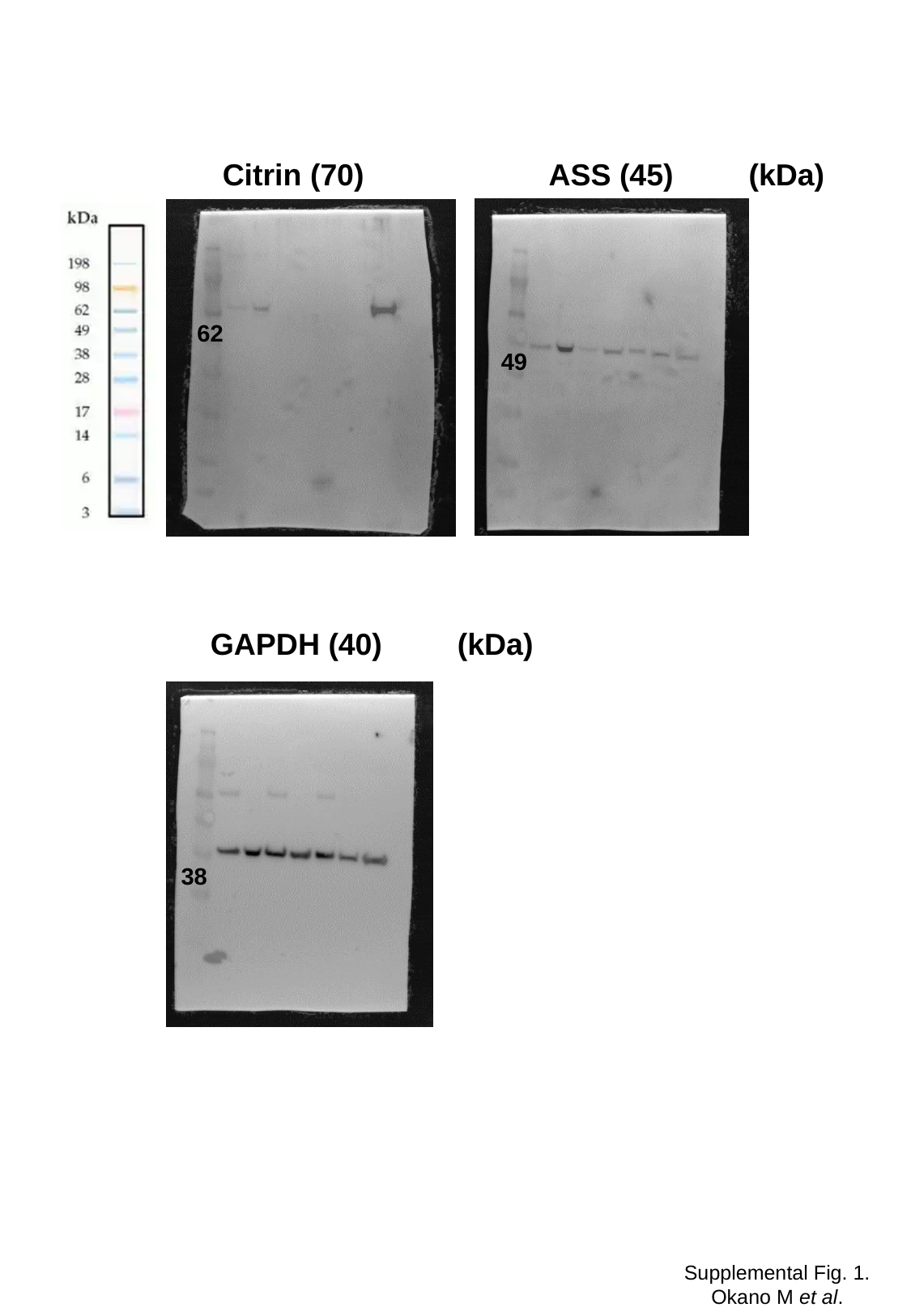

Citrin (70)　　　 ASS (45)　　(kDa)
62
49
GAPDH (40)　　(kDa)
38
Supplemental Fig. 1. Okano M et al.

## Slide 3
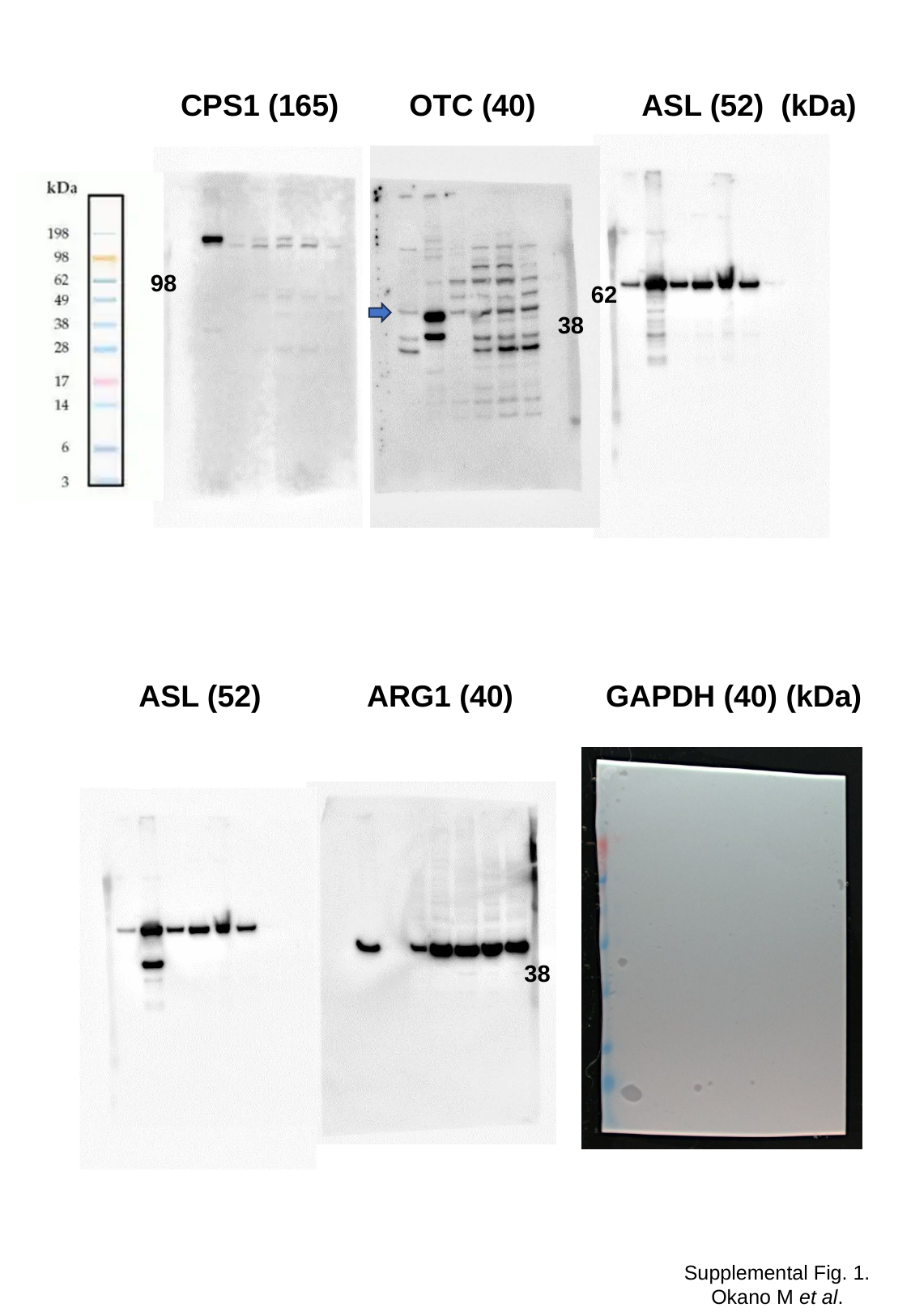

CPS1 (165) 　OTC (40)　　　ASL (52) (kDa)
98
62
38
ASL (52)　　　ARG1 (40)　 GAPDH (40) (kDa)
38
Supplemental Fig. 1. Okano M et al.
